# Supplementary material for: A set of multi-entry identification keys to African frugivorous flies (Diptera, Tephritidae)
Source: Zookeys. 2014 Jul 24;(428):97–108. doi: 10.3897/zookeys.428.7366 (PMC4143993; doi:10.3897/zookeys.428.7366)
Supplement: Supplementary material 6 — Key to Ceratitis [file zookeys-428-097-s006.zip › SF6_ZooKeys_key to Ceratitis/key/SF6_ZooKeys_key to Ceratitis/Media/Html/Ceratitis querita .htm]

Ceratitis (Pterandrus) querita (Munro)


***Ceratitis*** **(*Pterandrus*) *querita* (Munro)**

*Trirhithrum queritum* Munro, 1937: 10.

Body
length: 3.90 (3.80-4.20) mm; wing length: 4.15 (4.00-4.40) mm.

Male

Head: Antenna orange-brown. First flagellomere 2-3 times as
long as pedicel. Arista with short rays; basoventral rays shorter and sparser
than basodorsal rays. Frons convex; yellow, sometimes with darker patches; with
short scattered setulae darker than frons. Frontal setae well developed.
Frontofacial angle with brown band. Face white; gena brown. Genal seta and
setulae black, well developed. Occiput mainly pale.

Thorax:
Postpronotal lobe dark yellow, with black spot. Scutal pattern, shiny black,
center with silvery microtrichia; with pale and dark pilosity; no streaks or
spots, except very small white separate prescutellar spots. Scapular setae
dark. Scutellum yellow, basal spots well developed and joined, occupying full
anterior margin of scutellum; apical spots extending to basal 0.33 almost
touching basal spots, joined, weakly incised. Anepisternum on ventral half dark
brown, dorsal half paler brown, demarcated with pale line dorsally and
ventrally; setulae black.

Legs:
Yellow, femora dark brown; midtibia and hindtibia brown on basal two-third. No
feathering, pilosity and ventral setae on forefemur black.

Wing:
bands brown. Marginal band forming continuous band with anterior part of discal
band; cubital band joining junction of marginal and discal bands; medial band
absent; crossvein R-M opposite middle of discal cell. Apex of vein R_1_
distal to level of crossvein R-M. Crossvein DM-Cu oblique anterobasally.

Abdomen:
Shiny black, except tergite 1 yellowish orange. Tergites 2 and 4 with silvery
band along respectively posterior half and almost entire width. Setation and
banding typical for subgenus. Male epandrium in lateral view distinctly
broadened; posterior lobe of lateral surstylus slender, apical end curved.

Female

As
in male. Ovipositor shorter than preabdomen. Aculeus at least 10 times longer
than wide; tip distinctly broadened, and lateral margin straight.

(Description
after De Meyer & Freidberg, 2006)
